# Targeted therapy of multiple myeloma

**DOI:** 10.37349/etat.2021.00057

**Published:** 2021-10-31

**Authors:** Shan Zhou, Renxi Wang

**Affiliations:** Beijing Institute of Brain Disorders, Laboratory of Brain Disorders, Ministry of Science and Technology, Collaborative Innovation Center for Brain Disorders, Capital Medical University, Beijing 100069, China; Institute of Oncology Research, Switzerland

**Keywords:** Multiple myeloma, targeted therapy, potential targets, immune-based therapies, precision medicine

## Abstract

Multiple myeloma (MM) is a malignant proliferative disease of monoclonal plasma cells (PCs) and is characterized by uncontrolled proliferation of PCs and excessive production of specific types of immunoglobulins. Since PCs are terminally differentiated B cells, the World Health Organization (WHO) classifies MM as lymphoproliferative B-cell disease. The incidence of MM is 6–7 cases per 100,000 people in the world every year and the second most common cancer in the blood system. Due to the effects of drug resistance and malignant regeneration of MM cells in the microenvironment, all current treatment methods can prolong both overall and symptom-free survival rates of patients with MM but cannot cure MM. Both basic and clinical studies have proven that targeted therapy leads to a clear and significant prolongation of the survival of patients with MM, but when the disease recurs again, resistance to the previous treatment will occur. Therefore, the discovery of new targets and treatment methods plays a vital role in the treatment of MM. This article introduces and summarizes targeted MM therapy, potential new targets, and future precision medicine in MM.

## Introduction

**Figure 1.**
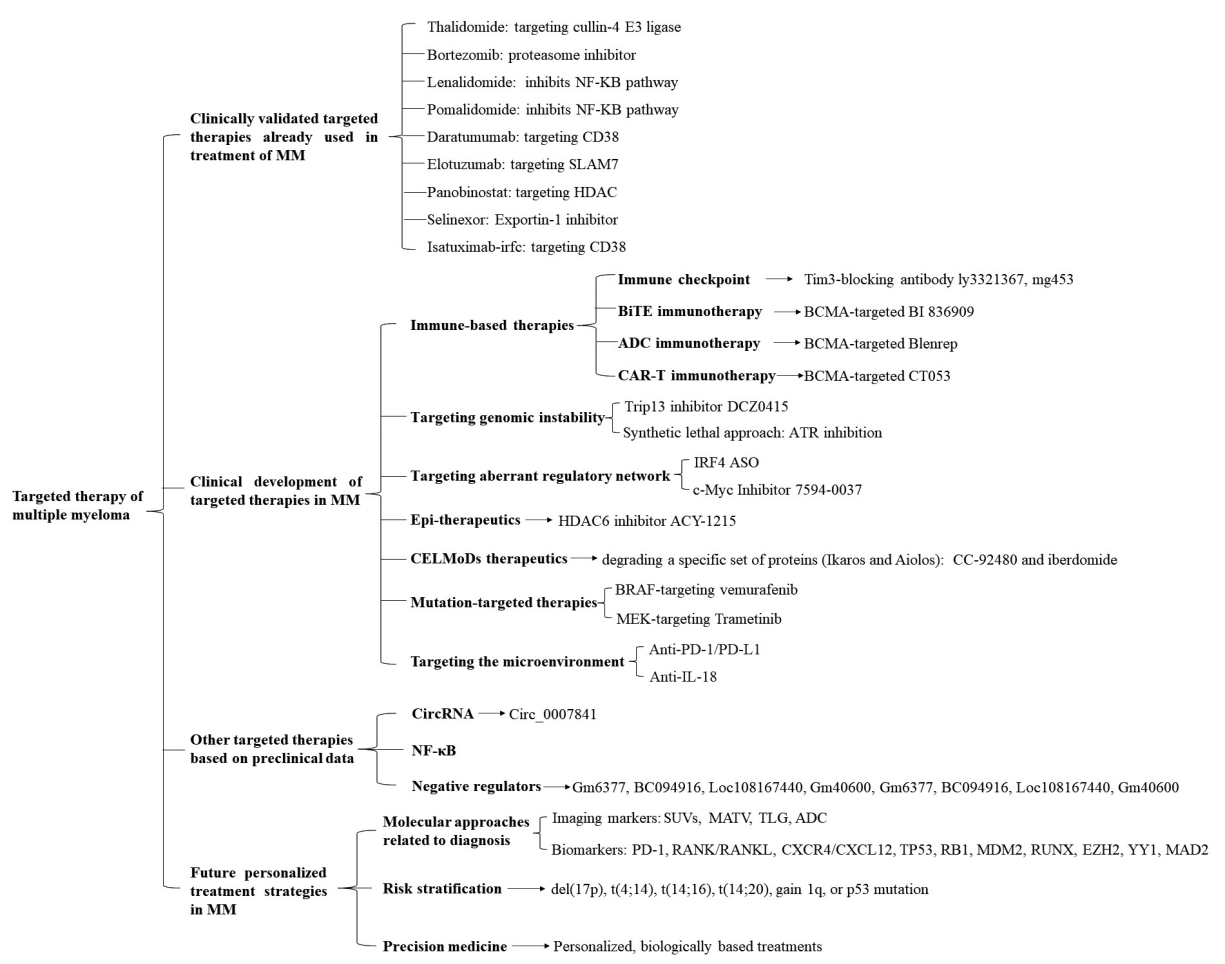
An overview of the different targeted therapies of therapeutic interest in MM. Targeted therapies are divided into 4 sections: 1. Clinically validated targeted therapies already used in treatment of MM including cullin-4 E3 ligase-targeted thalidomide, proteasome inhibitor bortezomib, NF-κB pathway inhibitor lenalidomide and pomalidomide, CD38-targeted daratumumab and isatuximab-irfc, SLAM7-targeted elotuzumab, HDAC-targeted panobinostat exportin-1 inhibitor selinexor; 2. Clinical development of targeted therapies in MM including immune-based therapies, targeting genomic instability, targeting aberrant regulatory network, epigenetic (Epi)-therapeutics, CELMoDs therapeutics, mutation-targeted therapies, targeting the microenvironment, and so on; 3. Other targeted therapies based on preclinical data including targeting CircRNA, NF-κB, negative regulators, and so on; 4. Future personalized treatment strategies in MM including molecular approaches related to diagnosis, risk stratification and precision medicine. SLAM7: signaling lymphocytic activation molecule family member 7; HDAC: histone deacetylase; Tim3: T cell immunoglobulin-3; BiTE: bispecific T-cell engager; BCMA: B-cell maturation antigen; ADC: antibody-drug conjugates; CAR-T: chimeric antigen receptor (CAR) T-cell (CAR-T); ATR: ataxia telangiectasia and Rad3 related; IRF4: interferon regulatory factor 4; ASO: antisense oligonucleotide; c-Myc: cellular myelocytomatosis; BRAF: v-raf murine sarcoma viral oncogene homolog B; MEK: mitogen-activated extracellular signal-regulated kinase; PD-1: programmed cell death-1; PD-L1: programmed death-ligand 1; IL-18: interleukin 18; Circ: Circular; SUVs: standard uptake values; MATV: metabolically active tumour volume; TLG: total lesion glycolysis; RANK: receptor activator of nuclear factor kappa B; RANKL: receptor activator of nuclear faktor kappa B ligand; CXCR4: C-X-C chemokine receptor type 4; CXCL12: C-X-C chemokine ligand 12; TP53: tumor protein p53; RB1: RB transcriptional corepressor 1; MDM2: mouse double minute 2; RUNX: runt-related transcription factor 1; EZH2: enhancer of zeste homolog 2; YY1: Yin Yang 1; CELMoDs: cereblon E3 ligase-modulating drugs

In terms of disease progression, multiple myeloma (MM) develops from a malignant precursor disease, progresses to monoclonal gammopathy of undetermined significance (MGUS), and then to smoldering MM (SMM) followed by active MM, which is often accompanied by anemia (usually the first symptom), bone pain (the most common symptom), renal insufficiency, infection, bleeding, neurological symptoms, hypercalcemia, amyloidosis, and other symptoms. Due to relapse after treatment, MM will progress to relapsed/refractory MM (RRMM). Most of these cases eventually develop into advanced plasma cell leukemia (PCL). At a molecular level, MM is caused by single or multiple gene mutations, heterotopic, chromosome changes, and other factors and mediated by abnormal chromosome hyper-diploidy, immunoglobulin heavy chain shift, cell cycle gene imbalance, nuclear factor-kappaB (NF-κB) pathway changes, and abnormal DNA methylation patterns [1]. In addition, it has been proven that MM cells also rely on the bone marrow (BM) microenvironment to support their growth, survival, and drug resistance. This interaction between cells and the microenvironment promotes the proliferation of tumor cells and weakens the immune surveillance and effector function of MM cells so that tumor cells can grow in an uncontrolled and unrestricted manner. Based on MM pathophysiology and the microenvironment, targeted therapies have been ongoing (Figure 1).

## Clinically validated targeted therapies already used in the treatment of MM

In the past, the Food and Drug Administration (FDA) approved some treatment drugs and modalities for MM (Table 1) [2–13]. In recent years, clinical guidelines have divided the treatment of MM into two categories: (1) those suitable for transplantation and (2) those not suitable for transplantation. For patients who are not suitable for transplantation, cytotoxic drugs are generally selected in combination with chemotherapy. These cytotoxic drugs generally have a slow onset, but relief from symptoms lasts a long time, and the time interval for suppressing recurrence is long, which can prolong the survival period of patients and delay the progression of clinical symptoms. For patients who are suitable for transplantation, targeted therapy is usually used clinically to enable the patient to undergo transplantation as soon as possible after remission. Generally, in patients who need BM transplantation, bortezomib (Velcade), lenalidomide (Revlimid), and dexamethasone (VRD) are used for about 3 to 4 treatment cycles before transplantation is performed. Patients who are not suitable for transplantation can also be treated with the above-mentioned drugs for 8 to 12 cycles, and then lenalidomide is used for maintenance treatment. Even though these treatments have a significant effect on prolonging the survival period of MM [14, 15], due to the heterogeneity of the sensitivity of patients to drugs and the need for long-term medication for patients with MM. Many patients have developed drug resistance to existing drugs, which leads to the recurrence of MM. The survival rate of recurrent MM is extremely low, so the discovery of new drug targets and the research of new therapies are urgently needed with respect to the treatment of MM.

**Table 1.** The current drugs and treatment methods for MM

**Years**	**Main drugs/treatment method**	**Composition or function**	**Application effect**
1960	Melphalan Cyclophosphamide corticosteroids	Alkylating agent	
1990	HSCT	Autologous hematopoietic stem-cell transplantation.	
1990	Thalidomide	Immunomodulatory; the first drug to show activity in RRMM; the main function is to suppress angiogenesis.	Toxic and side effects of the dose on peripheral nerves.
2003	Bortezomib	The proteasome inhibitor, which inhibits the NF-κB pathway, is often involved in the combined treatment of NDMM and RRMM and triggers “immunogenic” cell death, which can trigger effective anti-MM immune responses and disease control.	
2005	Lenalidomide	Immunomodulator that inhibits NF-κB pathway, activates caspase and promotes apoptosis [5]. It is the first-line drug for newly discovered MM. It is mainly involved in immune regulation, suppressing angiogenesis and proliferation.	Combined with bortezomib and dexamethasone (VRD), the 4-year overall survival rate was 81%. More than 2% of the patients had grade 3/4 adverse reactions: 63.7% of the patients had blood and lymphatic system diseases, and 13.7% had nervous system diseases [2].
2013	Pomalidomide	Immunomodulator inhibits NF-κB pathway, activates caspase, and promotes apoptosis [5].	Currently, it is approved to be combined with dexamethasone, elozumab, and dalatuzumab [3]. The median of PVD and VD was 11.2 and 7.1 (HR = 0.61, 95% CI: 0.49–0.77; *P* < 0.0001). Hematologic toxicity, gastrointestinal side effects, and rash are common.
2015	Daratumumab Elotuzumab	Monoclonal antibodies targeting CD38 and slamf7. Treatment of newly discovered and refractory MM.	D-RVd improved the complete remission rate and the minimal residual disease (MRD) negative rate, and the single drug remission rate was 29.2% [6]. Grade 3/4 neutropenia: d-RVd/RVD 41.4%, 21.6%; lymphopenia: 23.2%, 21.6%; thrombocytopenia: 16.2%, 8.8% [13].
2015	Panobinostat	Pan-HDAC inhibitor. For RRMM.	In combination with bortezomib and dexamethasone, the PFS was significantly improved, but the side effects were significant [11]. The overall response rate was 34.5%, the median time to response was 1.4 months [9]. Thrombocytopenia 63.6%, fatigue, and diarrhea 20.0%. The incidence of serious adverse reactions was 67.3% [9].
2019	Selinexor	Exportin-1 inhibitor specifically blocks xpo1 protein. For the treatment of RRMM, especially grade 3 refractory myelomas.	It is often combined with dexamethasone. The median duration of PFS was 13.93 months in the SVD group and 9.46 months in the VD group. This means that the median PFS increased by 4.47 months. Nausea, vomiting, loss of appetite. 73% of patients had thrombocytopenia [10].
2020	Isatuximab-irfc	The monoclonal antibody, targeting CD38, is used for RRMM. The immune effect function of MM was restored by blocking immunosuppressive T cells [4].	It was combined with pomalidomide/dexamethasone (PD). The median PFS of ISA-PD was 11.5 months, the median PFS of PD was 6.5 months, the HR was 0.60, the ORR of ISA-PD was 60.4%, and the ORR of PD was 35.3% [8]. In monotherapy, infusion response was 51%, fatigue 37%, nausea 32%, upper respiratory tract infection 24% and cough 23% [7]. Non-hematological adverse events caused by is a PD: fatigue 64%, ir40%, upper respiratory tract infection, and cough 40% [12].

HSCT: hematopoietic stem cell transplantation; PFS: progression-free survival; ORR: objective response rate; HR: hazard ratio; NDMM: newly diagnosed MM; PVD: pomalidomide + bortezomib (Velcade) + dexamethasone (DEX); VD: bortezomib (Velcade) + dexamethasone (DEX); D-RVd: daratumumab + lenalidomide (Revlimid) + bortezomib (Velcade) + dexamethasone (DEX); SVD: Selinexor + bortezomib (Velcade) + dexamethasone (DEX); ISA-PD: isatuximab with pomalidomide/dexamethasone; xpo1: exportin 1

## Clinical development of targeted therapies in MM

### Immune-based therapies

Immune-based therapies such as immune checkpoint Tim3 inhibitors, BiTE immunotherapy, ADC immunotherapy, and CAR-T (Table 2) immunotherapy have been becoming therapeutic hot-researches in MM.

**Table 2.** Immune-based therapies in MM

**Therapy**	**Structure**	**Trial stage**	**Dosage and effective rate**	**Adverse reactions**	**Approved usage**
Tim3 inhibitors such as LY3321367, MG453	The small molecular inhibitor	All of them have completed phase I clinical trials and have shown antitumor activity	Anti Tim3 drug tsr-022, Tim3 blocking antibody LY3321367, MG453		Tim3 antibody has been clearly expressed to improve AML
BiTE® BI 836909 [16–18]	Single-chain variable fragment (scFv) composed of anti-BCMA scFv at the N-terminal and anti-CD3ε scFv at the C-terminal, followed by hexahistidine	Animal experiment	0.5, 0.05, 0.005 mg/kg per day for 26 days; median survival was 43.5 days (0.5 and 0.05 mg/kg per day) and 43 days (0.005 mg/kg per day), whereas the control group was 34 days	Neurotoxicity	In 2014, FDA approved for the treatment of Philadelphia chromosome-negative relapsed/refractory B-cell precursor acute lymphoblastic leukemia [6]
Blenrep	Joint: non cleavable MC; Payload: MMAF	The first stage NCT020 64387, DREAMM-1 [19, 20]	4 mg/kg every 3 weeks ORR 60%; sCR 2 (6%), CR 3 (9%), VGPR 14 (40%); mPFS 12 months; mDOR 14.3 months	Grade 3 and 4 thrombocytopenia (35%), anemia (17%); hemocytopenia (52%); G1, 2 corneal events: blurred vision (52%), dry eyes (37%)	In early August 2020, it was approved in the United States for the treatment of relapsed or refractory MM in adult patients who have received at least four previous therapies, including anti-CD38 monoclonal antibody. In latter August 2020, Europe was also on trial
		The second stage, NCT03525678, DREAMM-2 [21]	2.5 or 3.4 mg/kg every 3 weeks; 2.5 mg/kg: ORR 30 (31%); sCR/CR 3 (3%), VGPR (15%); PD 56 (58%); mPFS 2.9 months; 3.4 mg/kg: ORR 34 (34%); sCR/CR 3 (3%), VGPR 17 (17%); PD 55 (56%); mPFS 4.9 months	Grade 3 and 4 keratopathy (27% in 2.5 mg/kg, 21% in 3.4 mg/kg), thrombocytopenia (20% and 33%) and anemia (20% and 25%)	
CAR-T: CT053 (NCT03716856, NCT03302403 and NCT03380039) [22]	It includes anti-BCMA human scFv, CD3 ζT cell activation domain, and 4-1BB costimulatory domain	Phase I clinical trial	CT053 was given once after fludarabine/cyclophosphamide treatment; the total remission rate was 87.5% and the complete remission rate was 70.8%	Hematological toxicity, including a 95.8% decrease in white blood cell count and 66.7% thrombocytopenia. One subject died of BM failure and neutropenia infection. There were 62.5% cytokine release syndrome and 12.5% neurotoxicity	FDA approved two anti-CD19 CAR-T cell products: Yescarta and Kymriah used to treat patients with recurrent and/or refractory NH [23], Kymriah was initially approved in the USA for relapsed or refractory pediatric or young adult B-cell precursor acute lymphoblastic leukemia in 2017, and subsequently approved for adults with relapsed or refractory diffuse large B-cell lymphoma in early 2018 [24]

AML: acute myeloid leukemia; sCR: stringent complete response; CR: complete response; VGPR: very good partial response; mPFS: median PFS; mDOR: median duration of response; MMAF: monomethyl auristatin F; MC: maleimidoca-proyl; DREAMM: the human study with belantamab mafodotin in patients with relapsed/refractory multiple myeloma; NH: non-Hodgkin lymphoma

#### Immune checkpoint

Immune checkpoint inhibitors have been launched in clinical practice to treat patients with several types of cancer, including MM [25]. Targeting the cytotoxic T lymphocyte antigen 4 (CTLA4) or PD-1/PD-L1 pathways checkpoint inhibitors have not been effective as a single agent in MM patients. In addition, current combinations of checkpoint inhibitors with immunomodulatory drugs have yielded disappointing results. However, checkpoint inhibitors remain an attractive therapy for MM [26]. T-cell immunoglobulin and Tim3 is an important negative regulator involved in cellular immunity. It is highly expressed in MM cells. In recent years, it has been found that Tim3 regulates the proliferation of MM cells through the NF-κB signaling pathway [phosphatidylinositol 3-kinase (PI3K), protein kinase B (PKB/AKT), mammalian target of rapamycin (mTOR), and NF-κB] [27]. Down-regulation of Tim3 expression can inhibit the proliferation of and induce apoptosis in MM cells and has a cumulative inhibitory effect on the NF-κB signaling pathway in conjunction with bortezomib [28]. In other words, if targeting Tim3 is successful in clinical trials, Tim3-targeted therapy can not only be used as a single therapy for MM but also can be combined with other drugs to increase drug efficacy. Therefore, Tim3 may become a new target for the treatment of NDMM/RRMM.

#### BiTE immunotherapy

BiTE has the specificity of binding double antigens, including an antibody that can bind to the antigen on T cells and an antibody that can bind to the tumor-specific antigen expressed on malignant cells. In this way, cytolytic synapses can be formed between T and malignant cells to enhance the interaction between T and malignant cells [16]. BiTE can activate and cause expansion of T cells by recognizing the CD3 subunit of T cells, produce cytokines, attack target cells, and finally lead to tumor cell lysis [17]. BiTE has the potential to be initiated immediately. BCMA, also known as tumor necrosis factor receptor (TNFR) superfamily 17 (SF17; TNFRSF17) or CD269, is a member of the TNFR superfamily [29]. It is a highly selective expression in normal plasmablasts (PBs) and plasma cells (PCs) but not in other tissues [30]. Of note, almost all MM cell lines express BCMA, and the expression of malignant PCs was higher than that of normal PCs [31]. BI 836909, a novel BCMA-targeted BiTE for the treatment of MM, induces highly specific and efficacious lysis of MM cells *in vitro* and shows anti-tumor activity *in vivo* (Table 2) [6, 18, 21, 32].

#### ADC immunotherapy

ADCs are monoclonal antibodies that bind drugs to tumor-associated antigen (TAA). Through the interaction of antigen and antibody, the drug binds to target cells expressing TAA. ADCs enter cells and release effective drug-induced toxicity to induce DNA damage and cell death. Cleavable junctions are treated by enzymes in target cells, while ADCs with non-cleavable junctions need to degrade the antibody in lysosomes to release the effective drug [19]. ADCs have two main advantages: (1) patients do not need to provide any of their own samples and personalized ADCs can be generated during treatment and (2) ADCs are in line with the requirements of modern medical development of precision medicine. Of course, some ADCs are cleaved outside the cells causing the premature release of drugs before entering the target cells, which will lead to the damage of other normal cells or tissues in the body and may also affect the function of other organs. The use of impermeable cell payloads (such as MMAF) or non-cleavable linkers can reduce the premature release outside the cells [17]. At present, blenrep (belantamab mafodotin-blmf), a BCMA-targeted ADC drug, has been approved for the treatment of highly refractory MM (Table 2) [20, 33, 34].

#### CAR-T immunotherapy

CAR-T cells are gene-modified T cells that express CAR specific to TAA. After binding, T cells are activated in a human leukocyte antigen (HLA)-independent manner [22, 24, 35, 36]. These CAR constructs are composed of single-chain antibodies (usually mouse or human) targeting TAA, which are connected to the CD3 ζ intracellular signal and costimulatory domains through extracellular regions and transmembrane domains.

The significant advantage of CAR-T cells is that these cells work for a long time. A single infusion alone may lead to persistent immunity to cancer cells. Compared with other treatments, the reduction in treatment times greatly reduces the damage of drugs or other substances to the body in the process of treatment. The disadvantage is that this type of drug requires a longer production time because it needs to go through the process of collecting white blood cells from patients for *ex vivo* expansion, transfer of autologous T cells, and reinfusion to patients, which is time-consuming. During long-term T-cell production, patients may develop leukopenia or other diseases that are caused by a decline in defense function. Of course, the immune relationship between reduced immune cells and CAR-T cell infusion may also lead to the progression and deterioration of related diseases. Another potential drawback is the use of pretreated lymphadenectomy. The decrease of endogenous lymphocyte level and the expansion of CAR-T cells may affect the rescue therapy after the failure of CAR-T cell therapy. CT053 is a BCMA-targeted CAR-T cell therapy and has become a breakthrough treatment in MM (Table 2) [23, 37, 38].

### Targeting genomic instability

There are currently several small molecule inhibitors targeting chromosomal instability such as poly(ADP-ribose) polymerase (PARP), Akt, Aurora kinase, and spindle kinase inhibitors which have been tested in mouse models and in early phase I/II trials [39]. Thyroid hormone receptor-interacting protein (Trip) 13 has been identified as a chromosomal instability gene and is associated with drug resistance, disease recurrence, and adverse prognoses in MM patients [40]. In animal experiments, a small molecule inhibitor (DCZ0415) of Trip13 can inhibit MM cell proliferation. It acts as a toxic agent of the spindle, which causes spindle multipole, destroys the repair of non-homologous end joining (NHEJ), inhibits the synthesis of DNA, and inhibits NF-κB activity in MM cells. More importantly, DCZ0415 works in conjunction with the melphalan and HDAC inhibitor panobinostat to suppress MM cell proliferation [41]. DCZ0415 also inhibits the protective effect of the BM microenvironment on myeloma cells [42]. Therefore, the discovery of more and new targets and new methods by targeting chromosomal instability such as Trip3 for the treatment of MM is important.

ATR inhibition, combined with piperlongumine, triggers synergistic MM cell cytotoxicity by enhancing oxidative stress while concomitantly blocking replicative stress response [43]. This novel combination targeted therapy, that is the synthetic lethal approach, provides an unmet medical need for a poor-prognosis subset of MM patients with extensive chromosomal instability and replicative stress [43].

### Targeting aberrant regulatory network

IRF4 can promote proliferation and survival of both MM stem and tumor cells, and the high level of IRF4 is directly related to the decline of overall survival rate in patients with MM [44]. The ASO of IRF4 inhibits the proliferation and survival of MM cells by reducing the expression of IRF4. IRF4 ASO monotherapy can prevent the formation of a xenograft tumor model and MM cell proliferation and lead to improvement in animal survival rates. In addition, IRF4 ASO presented more obvious advantages for MM treatment than drugs targeting other pathogenic molecules in MM in that it can eliminate the progenitor cells and malignant PCs of MM and retain normal hematopoietic stem cell development [45]. That is, if IRF4 ASO is successful, it may target disease cells more accurately and not cause much damage to other cells. Thus, side effects in MM patients may be greatly reduced. A study has shown that lenalidomide also causes down-regulation of IRF4 expression [46]. However, the use of selective IRF4 ASO may be superior to lenalidomide. ASO can be combined with MM drugs for treatment and has synergistic effects. Therefore, it is possible to study IRF4 ASO since IRF4 represents a potential therapeutic target of MM, especially with respect to the survival of patients who are difficult to treat with a standard of care drugs. Myelocytomatosis oncogene (MYC) is a direct target of IRF4 in activated B cells and myeloma. Critically, IRF4 was itself a direct target of MYC transactivation, generating an autoregulatory circuit in myeloma cells [47]. A novel c-Myc inhibitor, compound 7594-0037, can be used as a novel c-Myc inhibitor and is a potential candidate therapeutic drug for MM [48].

### Epi-therapeutics: a novel treatment modality in MM

Epigenetic aberrations are the pathogenic events that play important roles in leading to MM. HDACs influence the activity of several transcription factors disrupted in MM through histone modifications [49, 50]. Clinical trials have failed to show a clinical benefit with HDAC is as single-agent therapy. ACY-1215, a selective HDAC6 inhibitor, is a promising epigenetic targeted therapy for myeloma that has shown *in vitro* and *in vivo* effectiveness [51] and is now tested in two ongoing phases 1–2 clinical trials (ClinicalTrials.gov. NCT01323751 and NCT01583283) [52].

### CELMoDs

CELMoDs are larger molecules and work by quickly degrading a specific set of proteins (called Ikaros and Aiolos) [53–55]. They are potent modulators of the cereblon E3 ligase complex. They also stimulate the immune system and kill myeloma cells directly, even for myeloma that has become resistant to certain therapies. A new type of myeloma treatment called CC-92480 (a CELMoD) showed benefit in phase I international clinical trials [53–55]. It is synergistic with other therapies and has “excellent tumor penetration”. The other CELMoD currently in development is iberdomide [53–55].

### Mutation-targeted therapies

The mutational spectrum of MM with only a limited number of common recurrent mutations is now observed [56, 57]. Mutations in Kirsten rat sarcoma 2 viral oncogene homolog (KRAS), neuroblastoma RAS viral oncogene homolog (NRAS), and BRAF account for 50% of all cases at presentation and these numbers increase at relapse MM [58]. Inhibition of MEK, located downstream of the cellular homolog of viral raf gene (RAF) in the mitogen-activated protein kinases (MAPK) pathway, has emerged as a potential strategy to treat these patients [58]. Trametinib shows promise as a myeloma therapeutics based on responses seen in a heavily pretreated population [59]. BRAF-targeting vemurafenib has also been shown to result in responses. Targeting of the rat sarcoma (RAS)/MAPK pathway clearly could work in MM. However, it is compromised by intra-clonal and spatiotemporal heterogeneity. This will require changes to the way response is assessed, with markers directed to the subclone being treated becoming important [58].

### Targeting the microenvironment

BM microenvironment, composed of stromal cells, osteoblasts, osteoclasts, endothelial cells, immune cells, and a non-cellular compartment, provides the supportive role of malignant MM cell differentiation, migration, proliferation, survival, and drug resistance. Therefore, it is very interesting to target the BM microenvironment. Targeting specific niches within BM will have the potential for the treatment in MM and may facilitate earlier intervention to disrupt an environment that is permissive for myeloma progression [60]. PD-1 plays a critical role in the suppressive microenvironment of MM. Immunotherapies using anti-PD-1/PD-L1 strategies are promising treatment options for patients with MM [61]. The MM-niche-derived IL-18 drove the generation of myeloid-derived suppressor cells (MDSCs), leading to accelerated disease progression [62]. A preclinical study suggested that IL-18 could be a potential therapeutic target in MM [62].

## Other targeted therapies based on preclinical data

### CircRNA

Circ_0007841 has been shown to be up-regulated in BM biopsy cells from MM patients [63]. Circ_0007841 targets miR-129-5p and inhibit the effect of miR-129-5p, while miR-129-5p can inhibit the expression of jagged canonical Notch ligand 1 (JAG1) mRNA. Thus, circ_0007841 leads to MM cell proliferation, transformation, and drug resistance by promoting JAG1 overexpression. On the other hand, circ_0007841 gene knockout can inhibit the proliferation, metastasis, and drug resistance of MM cells by regulating the miR-129-5p/JAG1 axis. Another study found that circ_0007841 could also directly target miR-338-3p and cause acceleration of the progression of MM [64]. Circ_0007841 can up-regulate the expression of bromodomain-containing protein 4 (BRD4) by binding with miR-338-3p to promote the activation of PI3K/Akt signaling, thus promoting the proliferation and migration of MM cells. Therefore, circ_0007841 MM may become an important target for MM treatment in the future.

### NF-κB

NF-κB was previously one of the important targets for the treatment of MM. Five members in the NF-κB family have been found: (1) NF-κB1 (p50 and its precursor p105), (2) NF-κB2 (p52 and its precursor p100), (3) RelA (p65), (4) RelB and (5) c-Rel [65].

Two activation pathways of NF-κB have been demonstrated: 1) classical activation pathway, which is the main activation pathway of some receptors and cytokines. The extracellular signal factor binds to the receptor on the cell membrane to activate IkappaB (IκB) kinase (IKK). IKK phosphorylates the serine at the regulatory site of IκB subunit of NF-κB·IκB complex, which facilitates IκB subunit ubiquitination, which is then degraded by a protease and releases an NF-κB dimer. Free NF-κB will enter the nucleus and bind to the genes with NF-κB binding sites to initiate the transcription process and 2) non-classical activation pathway, mainly consisting of the p52/RelB dimer. The non-classical NF-κB kinase of the IKK complex selectively phosphorylates the p52 precursor, p100. Phosphorylation of p100 results in the removal of the C-terminal part of the protein (also known as IκBδ) and its degradation in the proteasome. This process produces a functional p52 protein, which can then dimerize with RelB and accumulate in the nucleus in which regulates its target gene. At least 17% of primary MM cells and 42% of MM cell lines were found to have mutations in several major components of the NF-κB pathway. Although the NF-κB activation pathway is well known and has led to the production of corresponding drugs, the change of new genes and the activation of new sub-pathways in this pathway will become a new target for MM treatment.

### Negative regulators or tumor-suppressors in PBs and PCs

MM characterized by cancerous proliferation of PBs and PCs remains incurable in many patients. Differentially-expressed molecules in MM PCs versus healthy PCs have been explored in order to identify novel targets for treating MM. Some molecules such as BCMA, are highly expressed in cancerous PBs and PCs and have been used as the key targets in the treatment of MM. Apart from positive regulators, negative regulators or tumor-suppressors have also been implicated to be potential biomarkers and therapeutic targets in some cancers [66, 67]. Some tumor suppressors such as down-regulation of candidate 2 (CECR2), contribute to tumor growth in laryngeal squamous cell carcinoma [68]. We searched for novel MM therapeutic targets by comparing mRNA expression patterns between the *Mus* musculus (mouse) myeloma PB-like myeloma SP 2/0 cell line and lipopolysaccharide (LPS)-induced PB/PC. We found that some molecules, such as Gm6377 and BC094916, were decreased in both normal PCs/PBs and SP 2/0 cells [69, 70]. Specifically, some molecules, such as Loc108167440 and Gm40600, were down-regulated in SP 2/0 cells but not in normal PCs and PBs [71, 72]. Of note, the overexpression of reduced forms of molecules, such as Gm6377, BC094916, Loc108167440, and Gm40600 suppressed SP 2/0 cell proliferation and suppressed tumor progression in the SP 2/0 xenograft mouse model [69–72]. These results suggest that modulation of negative regulators or tumor-suppressors may provide a potential therapeutic method for treating MM.

## Future personalized treatment strategies in MM

### Molecular approaches related to diagnosis of MM

Recent advances in the treatment of MM have pointed to accurate diagnosis of MM. It is possible that a better understanding of the genetic and epigenetic interactions in MM may reveal a new understanding of MM pathogenesis, new disease biomarkers, and hopefully the development of novel and a more effective diagnosis method. Present studies are evaluating a combined use of “imaging markers”, such as SUVs, MATV, TLG, ADC from positron emission tomography (PET), and magnetic resonance imaging (MRI) techniques respectively, and several “biomarkers” spanning from chemokine immune-modulators, such as PD-1, RANK/RANKL, CXCR4/CXCL12 to transcription factors, such as TP53, RB1, MDM2, runt-related transcription factor (RUNX) family, EZH2, YY1, mitotic arrest deficient 2 (MAD2) [73]. Imaging markers are used to characterize both tumor heterogeneity and metabolic data, whereas biomarkers may improve diagnosis and prognosis leading to precision medicine [73]. Although imaging and molecular integration could allow both early diagnosis and stratification of cancer prognosis, large-scale clinical trials will be necessary to translate pilot studies in the current clinical setting [73].

### Risk stratification in MM

The presence of del(17p), t(4;14), t(14;16), t(14;20), gain 1q, or p53 mutation is considered high-risk in MM [74]. The presence of any two high-risk factors is considered double-hit myeloma; three or more high-risk factors are triple-hit myeloma [74]. Standard risk patients can opt for delayed autologous stem cell transplantation (ASCT) at first relapse. Patients not candidates for transplant are treated with lenalidomide (Revlimid) plus dexamethasone (DEX, RD) until progression, or alternatively, a triplet regimen such as VRD for approximately 12–18 months. After ASCT, lenalidomide maintenance is considered for standard-risk patients especially in those who are not in VGPR or better, while maintenance with a bortezomib-based regimen is needed for patients with the intermediate or high-risk disease [75].

### Precision medicine in MM

A better understanding of diagnosis and risk stratification in MM will further inspire the development of precision therapy for MM. To date, the choice of therapy for an individual MM patient has been based on clinical factors such as age and comorbidities [76]. The widespread evolution, validation, and clinical utilization of molecular technologies, such as fluorescence *in situ* hybridization and next-generation sequencing has enabled the identification of a number of prognostic and predictive biomarkers for PFS, overall survival, and treatment response [76]. This will improve myeloma patient outcomes incorporating such biomarkers into the routine diagnostic workup of patients will allow for the use of personalized, biologically-based treatments [76].

## Conclusions

Due to the heterogeneity and resistance of MM patients to a variety of drugs, only a few drugs or limited targeted treatment can produce remission for patients with MM. In addition, the limitation of drug types makes it impossible to effectively treat patients with MM-associated drug resistance. Therefore, the discovery of new targets is undoubtedly the best hope for patients with MM, especially for patients with RRMM. Because any single pathway may have different side effects and the regulation of a single molecule is prone to drug resistance, many single molecule-driven drugs need to be combined with other drugs in order to effectively conduct symptomatic treatment. However, due to cumulative toxic and side effects of multi-drug combination, it is still difficult to achieve precise treatment and targeted therapy. Among these newly discovered targets, BCMA is now the most popular because it targets the PCs of MM and reverses the damaged hematopoietic microenvironment. Moreover, BCMA does not induce drug resistance easily because it plays an important role in the survival of normal long-lived PCs. Importantly, BCMA-based therapy has received good clinical feedback, so it is the most promising new target for the effective treatment of MM. In the near future, we hope that more targets similar to BCMA will become new therapies for the treatment of MM. In the present, incorporating the molecular approaches related to diagnosis and risk stratification into the routine diagnostic workup of patients has been allowing for the use of personalized, biologically-based treatments in MM.

## Abbreviations

ADC: antibody-drug conjugates
ASO: antisense oligonucleotide
BCMA: B-cell maturation antigen
BiTE: bispecific T-cell engager
BM: bone marrow
BRAF: v-raf murine sarcoma viral oncogene homolog B
CAR: chimeric antigen receptor
CAR-T: chimeric antigen receptor T-cell
CELMoDs: cereblon E3 ligase-modulating drugs
Circ: circular
c-Myc: cellular myelocytomatosis
CR: complete response
DREAMM: the human study with belantamab mafodotin in patients with relapsed/refractory multiple myeloma
D-RVd: daratumumab + lenalidomide + bortezomib + dexamethasone
FDA: Food and Drug Administration
HDAC: histone deacetylase
HR: hazard ratio
IKK: IkappaB kinase
IL-18: interleukin 18
IRF4: interferon regulatory factor 4
ISA-PD: isatuximab with pomalidomide/dexamethasone
IκB: IkappaB
MDM2: mouse double minute 2
MM: multiple myeloma
MMAF: monomethyl auristatin F
mPFS: median progression-free survival
NDMM: newly diagnosed multiple myeloma
NF-κB: nuclear factor-kappaB
ORR: objective response rate
PBs: plasmablasts
PCs: plasma cells
PD: pomalidomide/dexamethasone
PD-1: programmed cell death-1
PD-L1: programmed death-ligand 1
PFS: progression-free survival
RRMM: relapsed/refractory multiple myeloma
RUNX: runt-related transcription factor 1
scFv: single-chain variable fragment
sCR: stringent complete response
TAA: tumor-associated antigen
Tim3: T cell immunoglobulin-3
Trip: thyroid hormone receptor-interacting protein
VD: bortezomib + dexamethasone
VGPR: very good partial response
VRD: bortezomib, lenalidomide, and dexamethasone

## Declarations

### Copyright

© The Author(s) 2021.
